# Synergistic Effects of Biomimetic Structures on Heat Transfer Enhancement and Flow Resistance Reduction

**DOI:** 10.3390/biomimetics11030198

**Published:** 2026-03-09

**Authors:** Kaichen Wang, Yan Shi, Junjie Chen, Yuchi Dai

**Affiliations:** 1 School of Mechanical and Electric Engineering, Changchun University of Science and Technology, Changchun 130022, China; wkc1400@163.com (K.W.); chenjj202505@163.com (J.C.); custlaser_dyc@163.com (Y.D.); 2 National Base of International Science and Technology Cooperation for Optics, Changchun 130022, China

**Keywords:** biomimetic groove structure, heat transfer enhancement, performance evaluation criterion

## Abstract

This study numerically investigated the thermal performance of a rectangular channel incorporating scale-inspired biomimetic protrusion structures with micro-grooves on their surfaces. A three-dimensional numerical model was established and validated against experimental data under identical geometric parameters and boundary conditions, demonstrating good agreement in terms of outlet temperature and pressure drop over a wide range of Reynolds numbers. The effects of groove depth on friction factor, Colburn factor, and overall performance evaluation criterion (PEC) were systematically analyzed to elucidate the underlying flow and heat transfer mechanisms. The results indicated that the introduction of biomimetic grooves significantly modified the flow structure and thermal boundary layer development, thereby enhancing fluid mixing and heat transfer. However, excessive groove depth intensified flow separation and pressure loss, leading to performance deterioration. An optimal groove depth of 0.6 mm (approximately 40% of the fin height) was identified, which achieved the best balance between heat transfer enhancement and flow resistance. The findings provide theoretical guidance for the biomimetic surface design of high-efficiency heat exchangers.

## 1. Introduction

With the rapid development of high-power energy systems and advanced thermal management technologies, compact heat exchangers, owing to their small volume, low weight, and high heat transfer efficiency [1,2,3], were widely applied in aerospace engineering, advanced energy conversion systems, hydrogen energy utilization, electronic equipment cooling, and automotive thermal management. However, due to the inherent size constraints of compact heat exchangers, the simple installation of heat-conducting fins, although capable of increasing the contact area between the fluid and flow channels and thereby enhancing heat transfer performance, also intensified turbulence and reduced the fluid velocity within the channels [4,5]. Consequently, achieving high heat transfer efficiency while maintaining low flow resistance remained a critical challenge in the design and optimization of compact heat exchangers.

Biomimetics has been widely applied in fields such as robotics, materials science, mechanical engineering, aerospace engineering, biomedical engineering, and advanced manufacturing [6]. Notably, the application of biomimetic concepts to guide the design of a compact heat exchanger was regarded as a highly promising approach [7,8,9,10]. To enhance heat transfer performance, Goh et al. [11] arranged inverted fish-scale structures inside smooth circular tubes. Their results indicated that this configuration simultaneously enlarged the effective heat transfer area and disrupted the development of the thermal boundary layer. Compared with the smooth circular tube microchannel, the circular tube microchannel equipped with inverted fish-scale structures exhibited an increase of 111.2% in the average convective heat transfer coefficient. Luo et al. [12] investigated the effects of microchannel walls featuring leaf-vein-like structures on fluid flow and heat transfer characteristics. Although a certain degree of mass transfer capability was sacrificed, the heat transfer enhancement was substantial, indicating favorable application potential. Specifically, the flow resistance and heat transfer efficiency of the leaf-vein biomimetic microchannel were 60.15% and 44.89% higher, respectively, than those of the smooth circular tube microchannel. To improve the heat transfer performance of the gas cooler in transcortical CO_2_ heat pump systems, Li et al. [13] proposed a novel biomimetic honeycomb fractal heat exchanger and employed computational fluid dynamics (CFD) to investigate the conjugate heat transfer characteristics between supercritical CO_2_ and water. The results demonstrated that, compared with printed circuit heat exchangers, the biomimetic heat exchanger achieved improvements of 144.6% in the heat transfer coefficient and 20.4% in overall performance, while the multilevel fractal structures inside the exchanger further contributed to significant enhancement of comprehensive performance. To reduce energy consumption and improve energy utilization efficiency in data centers, Huang et al. [14] proposed a single-phase immersion cooling system integrated with latent heat thermal energy storage (LHTES) devices, in which a palmate leaf-shaped fin LHTES device was designed based on biomimetic principles to facilitate waste heat recovery. The results indicated that the biomimetic LHTES device outperformed conventional configurations in terms of charging–discharging performance and techno-economic efficiency, and could effectively reduce annual energy consumption and shorten the payback period under appropriate operating conditions and arrangements, thereby demonstrating strong engineering application potential. At present, the feasibility of employing biomimetic design concepts to enhance the heat transfer performance of heat exchangers has been well validated [15,16,17,18,19].

Among various biological surfaces, shark skin has attracted sustained attention due to its outstanding drag-reduction and heat transfer characteristics [20,21,22,23]. Walsh et al. [24] experimentally demonstrated at an early stage that micro-grooves aligned with the mainstream direction could significantly reduce wall frictional resistance. Wang et al. [25] systematically reviewed riblet–groove structures inspired by shark skin denticles and elucidated their underlying mechanisms in turbulence control and drag reduction, including the secondary vortex theory, height theory, and air-film theory, thereby providing new pathways for energy-efficient design. Fu et al. [26] focused on marine drag-reduction technologies and analyzed the influence of the number of riblets on performance. Their study highlighted the importance of the “area factor” in shear stress evaluation and identified riblet surface area as a key contributor to drag-reduction effectiveness. Although the complex microstructures of shark skin still posed manufacturing challenges for precise replication [27], their remarkable effectiveness in reducing fluid resistance and flow-induced noise had been widely validated [28,29]. Drag reduction was typically accompanied by increased flow velocity and coolant mass flux, which further enhanced the overall heat dissipation capability.

Based on this background, the present study proposes a biomimetic protrusion structure inspired by shark placoid scales and incorporates it into a rectangular channel to explore its synergistic effects on heat transfer enhancement and flow resistance reduction. A numerical simulation approach is employed to systematically investigate the influence of key geometric parameters on flow structure, vortex evolution, pressure drag characteristics, and heat transfer performance within the channel, with particular emphasis on the role of micro-groove depth. The study reveals the regulatory role of groove depth on flow separation and thermal boundary layer development, and identifies an optimal groove depth that achieves the best balance between heat transfer enhancement and flow resistance. This work provides new theoretical insights and geometric guidelines for the biomimetic surface design of high-efficiency compact heat exchangers.

## 2. Establishment and Validation of the Physical Model

### 2.1. Model Description

**Figure 1:**
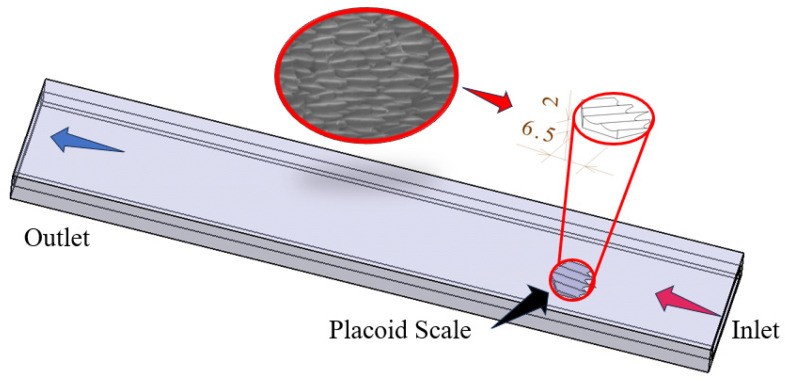
Single-fin channel model.

The investigation was conducted in a rectangular channel. The computational domain was designed according to the following criteria: ensuring fully developed inflow at the inlet, fully developed flow at the outlet, and minimizing the influence of the sidewalls and top wall on the flow and temperature fields in the vicinity of the fin. The finalized computational domain, as illustrated in Figure 1, had a length of L=200mm, a height of H=6mm, and a width of W=30mm.

**Figure 2:**
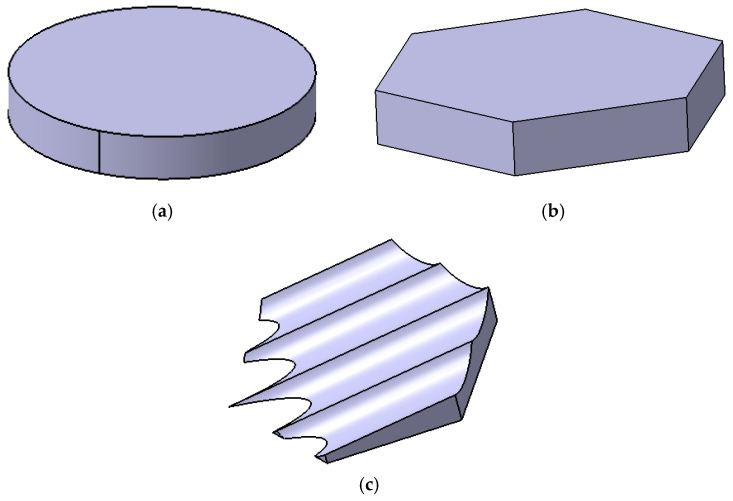
Geometric details of a single biomimetic unit: (**a**) cylindrical, (**b**) hexagonal prism, and (**c**) biomimetic placoid-scale fin with micro-grooves (groove depth = 0.6 mm, inclination angle = 15°).

The biomimetic shark placoid scale structure designed in this study was inspired by both the macroscopic morphology of shark skin denticles and their surface micro-riblet features [30,31]. The primary feature is a fin-like protrusion on the channel wall, and the secondary feature consists of micro-grooves engraved on the surface of this protrusion. During the design process, a streamlined base was first established as the base unit. Subsequently, to emulate the riblet characteristics on the placoid scale surface, four inclined micro-grooves were engraved on the surface of the base, forming a fin-like protrusion with a structural height of h=2mm, as shown in Figure 2c. These grooves are oriented along the flow direction and inclined upward to act as vortex generators.

This composite configuration was intended to mitigate large-scale flow separation through its streamlined base while simultaneously employing the inclined micro-fins as vortex generators to actively induce organized secondary flow structures and streamwise vortices in the near-wall region. Through this synergistic mechanism, the potential for coupled enhancement of heat transfer and flow control was systematically investigated.

To enable a systematic comparison and elucidate the intrinsic effects of different geometric configurations on flow and heat transfer characteristics, four representative fin geometries were selected in this study: a cylindrical fin, a hexagonal prism fin, a biomimetic placoid-scale fin, and a simplified ramp structure derived from the placoid-scale geometry. These configurations, respectively, represented typical features of streamlined bodies, prismatic bodies, and biomimetic composite structures. To ensure fairness in the comparison, the projected area of all configurations normal to the flow direction was maintained identical, thereby eliminating size effects arising solely from differences in frontal area.

In the specific design, the cylindrical fin (diameter D=13mm, height h=2mm) was employed as a classical streamlined benchmark case. Its wake flow behavior and heat transfer characteristics had been extensively investigated in the literature, providing a reliable reference for the present study. The hexagonal prism fin (with a circumscribed circle diameter equal to the cylinder diameter D and a height of h=2mm) represented a class of prismatic structures with fixed flow separation points, in which sharp edges induced distinct vortex shedding patterns and were commonly used to explore the relationship between separated flow and heat transfer enhancement. All geometric models were constructed using CATIA to ensure dimensional accuracy. For the biomimetic placoid-scale fin, the base width is 2 mm, the total height is 1.5 mm, and the groove depth is 0.6 mm (or 1.2 mm for Structure 3). The grooves are inclined at an angle of 9° relative to the base plane.

### 2.2. Governing Equations Creation and Boundary Conditions Setting

In the simulations, the fluid domain was treated as incompressible, and the flow and heat transfer processes were solved using the steady-state incompressible Navier–Stokes equations. The governing equations were formulated under the following assumptions: (1) the flow was steady; (2) the flow was three-dimensional and incompressible; and (3) the thermophysical properties of the fluid remained constant. Accordingly, the governing equations were expressed as follows [32]:

(1) $$\frac{\partial }{\partial x_{i}}\left(\rho u_{i}\right)=0$$

Momentum equation:

(2) $$\frac{\partial }{\partial x_{j}}\left(\rho u_{i}u_{j}\right)=-\frac{\partial \rho }{\partial x_{i}}+\frac{\partial \rho }{\partial x_{j}}\left[\mu \left(\frac{\partial u_{i}}{\partial x_{j}}+\frac{\partial u_{j}}{\partial x_{i}}-\frac{2}{3}\delta_{ij}\frac{\partial u_{k}}{\partial x_{k}}\right)\right]-\frac{\partial }{\partial x_{j}}\left(-\rho \bar{u_{i}^{\prime }u_{j}^{\prime }}\right)$$

Energy equation:

(3) $$\frac{\partial }{\partial x_{i}}\left(\rho u_{j}T\right)=\frac{\partial }{\partial x_{j}}\left[\left(\frac{\mu }{\mathrm{Pr}}+\frac{\mu_{t}}{\sigma_{T}}\right)+\frac{\partial T}{\partial x_{j}}\right]+S_{T}$$

This study employed the k−ω SST model, and the relevant governing equations were expressed as follows:

Turbulent kinetic energy k equation:
  (4) $$\frac{\partial (\rho k)}{\partial t}+\frac{\partial \left(\rho ku_{j}\right)}{\partial x_{j}}=\frac{\partial }{\partial x_{j}}\left[\left(\mu +\sigma_{k}\mu_{t}\right)\frac{\partial k}{\partial x_{j}}\right]+P_{k}-\rho \beta^{*}\omega k$$
  Specific dissipation rate ω equation:
  (5) $$\frac{\partial (\rho \omega )}{\partial t}+\frac{\partial \left(\rho u_{j}\omega \right)}{\partial x_{j}}=\frac{\partial }{\partial x_{j}}\left[\left(\mu +\sigma_{\omega }\mu_{t}\right)\frac{\partial \omega }{\partial x_{j}}\right]+P_{\omega }-\rho \beta \omega^{2}+2(1-F_{1})\frac{\rho \sigma_{\omega 2}}{\omega }\frac{\partial k}{\partial x_{j}}\frac{\partial \omega }{\partial x_{j}}$$
  The turbulent viscosity $\mu_{t}$ was expressed as:
  (6) $$\mu_{t}=\rho C_{\mu }\frac{k^{2}}{\varepsilon }$$
  (7) Re=uρDeμ
  (8) $$D=\frac{2\left(W\times H\right)}{\left(W+H\right)}$$

To quantitatively evaluate the heat transfer and flow performance of the channel, the following key dimensionless parameters were introduced:

The Nusselt number Nu is defined as:
  (9) $$\mathrm{Nu}=\frac{h_{\mathrm{air}}D_{e}}{\lambda }$$
  The Colburn factor j and the friction factor f of the channel are defined, respectively, as follows:
  (10) j=NuRe⋅Pr1/3
  (11) $$f=\frac{2\Delta {pA}_{c}}{\rho A_{0}u^{2}}$$
  The performance evaluation criterion PEC is defined as:
  (12) $$PEC=\frac{j_{b}/j_{0}}{{\left(f_{b}/f_{0}\right)}^{1/3}}$$

The subscript b denoted the channel with protruding structures (biomimetic channel), while the subscript 0 referred to the corresponding smooth reference channel under identical operating conditions. A performance evaluation criterion (PEC) value greater than unity indicated that the biomimetic channel exhibited superior overall heat transfer performance compared with the smooth channel under the same inlet velocity.

During the numerical simulations, appropriate boundary conditions were imposed to accurately represent the physical scenario. At the inlet, a velocity inlet boundary condition was specified, where the fluid entered the channel with a uniform temperature of T=333 K. The inlet velocity varied from 1.5 to 10 m/s, corresponding to a Reynolds number range of 1000–7000. At the outlet, an outflow boundary condition was applied, allowing fully developed flow to exit the domain naturally without backflow. The bottom wall temperature was maintained at a constant value of T=278 K. All remaining walls and extended sections were assumed to be adiabatic in order to minimize unintended heat transfer effects.

In the numerical model, the surfaces of the protruding structures were also treated as adiabatic. It should be noted that, in practical engineering applications, such protrusions are typically fabricated from materials with high thermal conductivity and may incorporate internal cooling channels, thereby enabling coupled enhancement of heat transfer through fluid flow and solid conduction. However, the primary objective of the present study was to isolate and quantitatively evaluate the fluid dynamic effects induced solely by the biomimetic geometry. Therefore, in both the current numerical model and the subsequent experimental model, the protrusions were simplified as adiabatic bodies to eliminate the influence of heat conduction on the analysis. A no-slip boundary condition was enforced on all channel surfaces and biomimetic structures, ensuring that the fluid velocity relative to the solid surfaces was zero. To further exclude the influence of cooling-induced temperature variations on the biomimetic structure walls and, consequently, on the overall heat transfer performance of the channel, the surfaces of the biomimetic structures were considered thermally insulated. The flow regime is determined based on both the Reynolds number and the geometric configuration. For the smooth channel, when Re<2000, the flow is treated as laminar and solved using the laminar Navier–Stokes equations. However, in channels featuring biomimetic protrusions, even at Reynolds numbers below 2000, the disturbances induced by the protruding structures promote early transition to turbulent flow. Therefore, the k–ω SST turbulence model is uniformly applied for all cases with protrusions across the entire Reynolds number range (Re<2000 and 3500<Re<7000). This parameter configuration enabled a comprehensive investigation of the effects of biomimetic structures on the heat transfer efficiency and flow characteristics within the precooler under different flow regimes.

The computational domain was discretized using high-quality structured hexahedral meshes. The core region of the mesh consisted of regular hexahedral elements with a characteristic size of 0.5 mm. To accurately capture near-wall flow phenomena, a dedicated boundary-layer mesh was generated, comprising four layers with a growth ratio of 1.2. Although four layers are relatively few, grid independence studies showed that further increasing the number of prism layers altered wall shear stress and heat flux by less than 2%, while substantially increasing computational cost. Therefore, the current four-layer setting is considered acceptable for balancing accuracy and efficiency. For future high-precision studies, a finer boundary layer mesh is recommended to better resolve near-wall flow details. To ensure that the dimensionless wall distance remained sufficiently close to unity under all simulated flow conditions, the height of the first layer was carefully adjusted according to the Reynolds number, such that the first-layer thickness was maintained within the range of 0.05–0.1 mm. A comprehensive assessment of mesh quality was conducted. The average element quality metric was 0.724, with a maximum value of 1.0. In terms of element distortion, the average aspect ratio was 2.453, while the maximum aspect ratio reached 10.598. All these metrics fell within the recommended ranges for CFD simulations, confirming that the overall mesh quality was sufficiently high to ensure accurate and reliable numerical results.

To eliminate the influence of mesh density on the simulation outcomes, a systematic grid independence study was first performed on the smooth channel, which served as a baseline for determining appropriate mesh parameters. The total number of mesh elements was progressively increased from an initial value of 6009 to a refined maximum of 74,646 elements. Key performance parameters, namely the Colburn factor and the friction factor, were compared across different mesh resolutions. The results indicated that the discrepancies between these parameters decreased gradually with increasing mesh density. Based on this convergence behavior and considering computational efficiency, a mesh containing approximately 12,009 elements was selected for the smooth channel. This mesh provided high resolution in critical regions, with 3436 elements allocated to the near-wall region, ensuring accurate resolution of boundary-layer phenomena.

The same meshing strategy—including element size, boundary-layer settings, and growth ratios—was then applied to channels with protrusion structures. Due to the geometric complexity introduced by the micro-grooves and inclined surfaces, additional boundary-layer refinement was required to accurately capture near-wall flow features. Consequently, the total mesh count for biomimetic channels ranged from 12,009 to 56,858 elements, depending on the specific configuration (e.g., groove depth, presence of grooves). The higher proportion of near-wall elements (up to 30% of total cells) is justified by the need to resolve detailed flow structures in the vicinity of the protrusions, which is critical for the reliable prediction of heat transfer and pressure drop.

## 3. Experimental Design

### 3.1. Setup of the Experimental System

**Figure 3:**
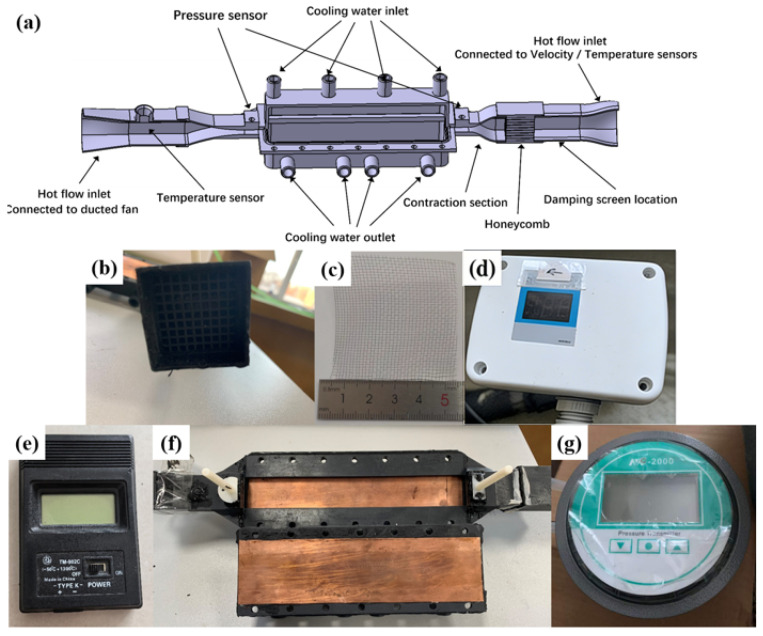
Schematic of the experimental System: (**a**) Overall Schematic of the experimental setup, (**b**) Honeycomb (**c**) Damping screen, (**d**) Velocity sensor, (**e**) T sensor, (**f**) Experiment section, and (**g**) Pressure transmitter.

The overall schematic of the experimental system is illustrated in Figure 3. The operating procedure of the experimental apparatus was described as follows. The airflow inside the duct was driven by a ducted fan located at the downstream end of the test rig. Upon activation, the fan generated a negative pressure within the system, thereby drawing ambient air into the channel from the left side of the experimental platform and establishing a controlled and stable airflow. By precisely adjusting the rotational speed of the ducted fan, the volumetric flow rate of air was continuously and steadily regulated.

An electrical resistance heater installed upstream of the test section inlet was employed to heat the incoming air. By finely modulating the electrical power supplied to the heater, the inlet air temperature was accurately controlled. Calibrated velocity and temperature sensors, located upstream of the test section inlet (as shown in Figure 3d and Figure 3e, respectively), were used to continuously monitor the inlet air velocity and temperature in real time. Prior to entering the test section, the airflow passed sequentially through a honeycomb flow straightener (Figure 3b) and a damping screen (Figure 3c). The honeycomb straightener served to break up large-scale vortices and align the flow, thereby effectively reducing intermittent turbulence and minimizing transverse velocity components. The damping screen further attenuated turbulence intensity, resulting in a more uniform and stable inflow. The conditioned airflow subsequently entered a carefully designed contraction section, which enabled uniform acceleration of the flow and ensured that the air entering the test section achieved the desired velocity profile. This contraction section also enhanced flow stability and reduced velocity fluctuations.

The detailed structure of the test section is shown in Figure 3f. After passing through the contraction section, the heated air entered the test section with a rectangular cross-section. The heat transfer surface of the test section was fabricated from a copper plate, the rear side of which contained cooling water channels. A large flow rate of temperature-controlled cooling water was circulated through these channels to maintain a constant wall temperature. The protruding structures were manufactured from polymethyl methacrylate (PMMA, commonly known as acrylic) using precision machining techniques. PMMA was selected primarily due to its extremely low thermal conductivity (approximately 0.2 W/m·K), which is nearly four orders of magnitude lower than that of the copper heat transfer surface (approximately 392 W/m·K). This material selection ensured that heat conduction through the protrusions was negligible, thereby confirming that any observed variations in heat transfer performance were predominantly attributed to flow modifications induced by the biomimetic protruding structures.

Pressure taps were installed at the inlet and outlet cross-sections of the test section, and a high-precision differential pressure sensor (Figure 3g) was used to measure the pressure drop across the test section. The main supporting structure of the rectangular channel was fabricated using photopolymerization-based 3D printing with photosensitive resin, while the heat transfer surface consisted of a 0.2 mm thick copper plate bonded to the structure. For performance comparison, a smooth channel without any protruding structures was also fabricated and tested as a reference case. As the air flowed through the test section, it was disturbed by the protruding structures, which enhanced convective heat transfer with the cooled wall. The outlet air temperature was measured at a stabilized region downstream of the test section outlet.

### 3.2. Uncertainty Analysis

Before formal experimental data acquisition, a strict instrument calibration procedure was carried out. All sensors, including those measuring temperature, velocity, and pressure, were zero-adjusted and calibrated prior to each experimental run. The differential pressure sensors and temperature sensors provided digital signal outputs. The minimum resolution of the differential pressure sensor was 1 Pa, while the temperature sensor had a resolution of 0.1 K.

To ensure that the system reached thermal equilibrium and a stable flow condition, the duct fan and electric heating wires were activated, and the system was pre-operated for at least 2 min. Data recording was initiated only after all monitored physical quantities (velocity, temperature, and pressure difference) became stable and their fluctuations were lower than the predefined threshold values. Under steady experimental conditions, five consecutive data sets were collected for each operating point, and their arithmetic mean was taken as the final result for subsequent analysis.

To evaluate the statistical reliability of the experimental results, each Reynolds number condition was independently repeated five times (n=5). The combined uncertainty of the experimental data $U_{C}$ consisted of the Type A uncertainty $U_{A}$, which originated from repeatability, and the Type B uncertainty $U_{B}$, which resulted from instrument accuracy. The combined uncertainty was calculated as:

(13) $$U_{C}=\sqrt{U_{A}^{2}+U_{B}^{2}}$$

Since the number of repeated experiments for each operating condition was 5 (less than 30), the Type A uncertainty $U_{A}$ was calculated using the expanded uncertainty method, expressed as:

(14) $$U_{A}=\frac{t}{\sqrt{n}}S$$

where t is Student’s *t*-factor corresponding to a 95% confidence level and the associated degrees of freedom. According to the distribution table, t=2776 was used for n=5 The parameter S represents the sample standard deviation of the five independent repeated measurements.

The Type B uncertainty $U_{B}$ was mainly determined by the sensor resolution and was calculated as:

(15) $$U_{B}=\frac{a}{k}$$

where a is the minimum resolution of the sensor. Assuming a uniform distribution of the measurement error, the coverage factor was taken as $k=\sqrt{3}$.

Based on the above methodology, the uncertainty analysis results for the outlet temperature $T_{\mathrm{out}}$ and pressure drop Δp are summarized in Table 1. The data in the table are presented in the form of “mean value ± expanded uncertainty.”

### 3.3. Numerical Validation

**Figure 4:**
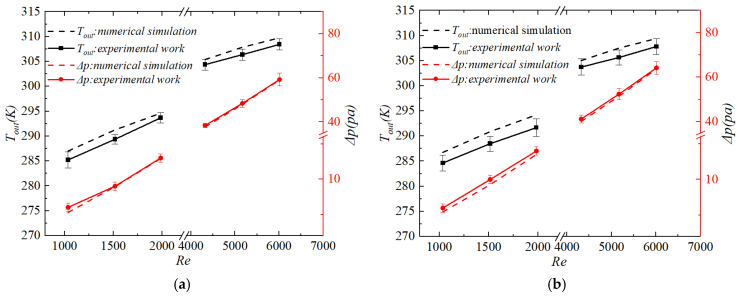
Comparison between Numerical Simulation Results and Experimental Data for Different Channels: (**a**) Smooth channel; (**b**) Biomimetic channel.

To verify the reliability of the established numerical model and solution methodology, numerical simulations were conducted using the same geometric parameters and boundary conditions as those employed in the experiments. Simulations were performed for both the smooth channel and the channel embedded with protrusion structures. The numerical simulation results are presented in Table 2. The simulated thermo-hydraulic characteristics, including the outlet temperature $T_{\mathrm{out}}$ and pressure drop Δp, were compared with the corresponding experimental measurements. The comparison results are presented in Figure 4. In this figure, the experimental data are represented by solid symbols and solid lines, whereas the numerical results are indicated by hollow symbols and dashed lines. The error bars denote the uncertainty range of the experimental data.

For the smooth channel, the comparison between numerical predictions and experimental data is shown in Figure 4a.

For the biomimetic channel, the corresponding comparison is illustrated in Figure 4b.

Quantitative statistical analysis shows that for the outlet temperature $T_{\mathrm{out}}$, the maximum percentage error between the numerical simulation and experimental measurement is 0.7% with an average percentage error of 0.5% for the smooth channel; for the biomimetic channel, the maximum percentage error is 0.9%, and the average percentage error is 0.7%. For the pressure drop Δp, the maximum percentage error is 12.0% with an average percentage error of 2.8% for the smooth channel, while the biomimetic channel has a maximum percentage error of 9.2% and an average percentage error of 4.2%.

The key comparison parameters were the outlet temperature $T_{\mathrm{out}}$ and the pressure drop Δp. It can be observed that the numerical results agreed well with the experimental measurements over the entire range of Reynolds numbers investigated. The deviations remained within an acceptable engineering error range. These results demonstrated that the numerical method, physical models, and meshing strategy adopted in this study provided reliable accuracy in predicting the thermo-hydraulic performance of heat exchanger channels. Therefore, the validated model was considered suitable for subsequent in-depth parametric studies and mechanism analyses.

## 4. Effect of Different Protrusion Structures on Flow Characteristics

### 4.1. Performance Comparison of Different Protrusion Structures

Compared with the smooth rectangular channel, the introduction of protrusion structures significantly altered the internal flow field and temperature distribution, thereby affecting the overall thermo-hydraulic performance. The protrusions induced flow separation, generated vortical structures, and disturbed the thermal boundary layer, which enhanced fluid mixing and consequently modified the momentum and heat transfer processes within the channel.

**Figure 5:**
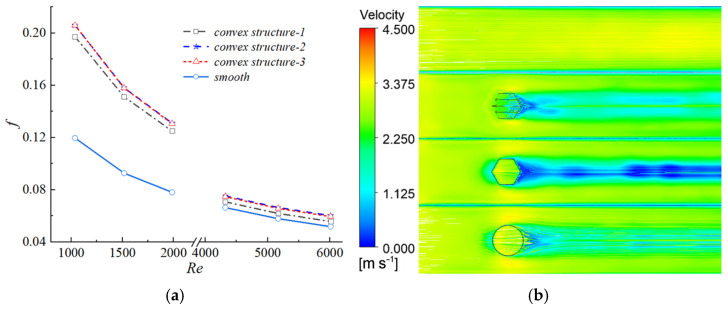
Friction Coefficient Variation and Corresponding Flow Field Characteristics: (**a**) Curves of friction factor f as a function of Reynolds number Re; (**b**) Velocity contours at the channel cross-section for Re = 1985.

To systematically compare the thermo-hydraulic performance, four channel configurations were investigated: the smooth channel without any protrusions (smooth), and three channels with different protrusion structures, namely the cylindrical protrusion (convex structure-3), the hexagonal prism protrusion (convex structure-2), and the biomimetic placoid-scale fin with micro-grooves (convex structure-1). Their geometric details are described in Section 2.1 and illustrated in Figure 2. As shown in Figure 5a, the friction factor of all channels decreased with increasing Reynolds number (Re). In both the laminar regime (Re < 2000) and the turbulent regime (Re > 3500), the f curves corresponding to the three protrusion structures nearly overlapped, exhibiting only minor differences. Quantitative analysis indicated that when Re < 2000, the friction factors of the protrusion channels were approximately 60–67.8% higher than that of the smooth channel. When Re > 3500, the increase was reduced to 6.6–16.2%. These results demonstrated that the introduction of protrusions significantly increased the additional flow resistance, with a more pronounced effect in the laminar regime. This behavior was mainly attributed to the disruption of laminar flow stability and the promotion of earlier flow transition induced by the protrusion structures. Figure 5b illustrates the velocity contours at the channel cross-section for Re = 1985 (laminar regime).

In the smooth channel, the streamlines remained nearly parallel, indicating a stable laminar flow pattern. After the introduction of protrusion structures, however, the originally parallel flow was significantly disrupted. The protrusions obstructed and redirected the incoming flow, inducing flow bifurcation and local acceleration, which consequently altered the velocity field distribution. Meanwhile, the wall shear stress increased markedly, leading to a rise in the friction factor of the biomimetic channel to 0.1249, which was substantially higher than that of the smooth channel (0.0781). In addition, relatively large low-velocity regions were observed downstream of all three protrusion configurations. Although these low-velocity zones exhibited similar overall shapes, noticeable differences existed in their internal flow structures due to variations in the flow disturbances induced by different protrusion geometries. These differences constituted one of the key factors contributing to the variation in heat transfer performance among the three configurations.

**Figure 6:**
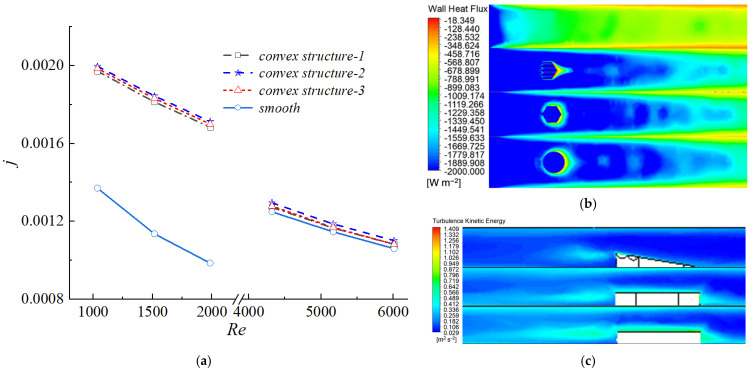
Analysis of heat transfer characteristics and flow mechanisms. (**a**) Variation of Colburn factor j with Re; (**b**) Contour map of wall heat flux; (**c**) Contour map of local turbulent kinetic energy.

Figure 6a presents the variation of the Colburn factor j with Reynolds number Re. Similar to the trend observed for the friction factor, the j factor of all channels decreased with increasing Re, and the curves corresponding to the three protrusion configurations nearly overlapped in both the laminar and turbulent regimes.

In the laminar regime (Re < 2000), the protrusion structures induced flow instability and promoted an earlier transition from laminar to turbulent flow, thereby significantly enhancing heat transfer performance. The wall heat flux contours shown in Figure 6b indicated that larger regions of high heat flux were formed downstream of the protrusions. As a result, the j factor of the biomimetic channels increased by approximately 43.8–73.8% compared with that of the smooth channel. Although the absolute value of the j factor decreased with increasing Re, the relative enhancement ratio compared with the smooth channel showed an increasing trend within this range.

In contrast, in the fully developed turbulent regime (Re > 3500), although the protrusion structures further intensified local turbulent kinetic energy, as illustrated in Figure 6c, the overall heat transfer enhancement became limited. Under these conditions, the j factor increased by only about 3.9% compared with the smooth channel. This indicated that, in a strongly turbulent background, the additional disturbances introduced by the protrusions yielded diminishing returns in terms of heat transfer enhancement.

**Figure 7:**
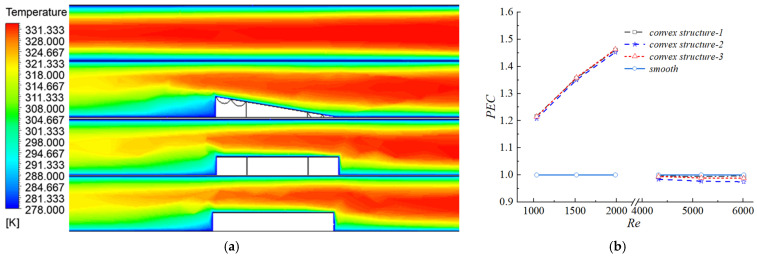
Temperature distribution and performance evaluation of heat transfer enhancement. (**a**) Temperature contour verifying the trend at Re = 1985; (**b**) PEC (performance evaluation criterion) of smooth and biomimetic channels over varying Re.

The temperature contours (Figure 7a) further confirmed the aforementioned trend. At Re = 1985, the outlet temperature of the biomimetic channel decreased to 294.0 K, whereas that of the smooth channel remained at 306.1 K. Local low-temperature regions were observed on the leeward side of each protrusion, but they had a limited effect on the overall temperature field. Figure 7b shows that the performance evaluation criterion (PEC) of the smooth channel remained constant at 1. When 1000 < Re < 2000, the PEC of the channels with protrusions exceeded 1, indicating that the heat transfer gain outweighed the pressure drop penalty at low Reynolds numbers. Conversely, at Re > 3500, the PEC dropped below 1 (approximately 0.99 for the scaled structure and below 0.98 for the others), suggesting that the increase in flow resistance dominated over the heat transfer enhancement at high Reynolds numbers.

The comprehensive analysis indicated that the scaled biomimetic structure exhibited pronounced flow and heat transfer enhancement at low Reynolds numbers, with its performance evaluation criterion (PEC) exceeding 1, demonstrating that the heat transfer gains outweighed the adverse effects of the increased pressure drop. However, under high Reynolds number conditions, although the scaled biomimetic structure still induced some turbulence and provided limited heat transfer enhancement, the significant increase in pressure drop led to a slightly lower overall performance compared to the smooth channel, with a PEC of approximately 0.99. Overall, the scaled biomimetic structure showed good potential for applications in low Reynolds number regimes, while under high Reynolds number conditions, its overall performance was similar to that of the smooth channel, indicating scope for further optimization and structural improvement.

### 4.2. Analysis of Flow Resistance Characteristics

To further analyze the components of flow resistance, the total friction factor of the channel (f) was decomposed into the viscous resistance coefficient ($f_{v}$) and the pressure drop resistance coefficient ($f_{p}$):

(16) $$f_{v}=\frac{8\tau_{w}}{\rho u^{2}}$$

(17) $$f_{p}=f-f_{v}$$

The wall shear stress $\tau_{w}$ is directly obtained from the CFD post-processing.

The contribution of the pressure drop resistance coefficient to the total resistance (ε) was defined as follows:

(18) $$\varepsilon =\frac{f_{p}}{f}*100\%$$

**Figure 8:**
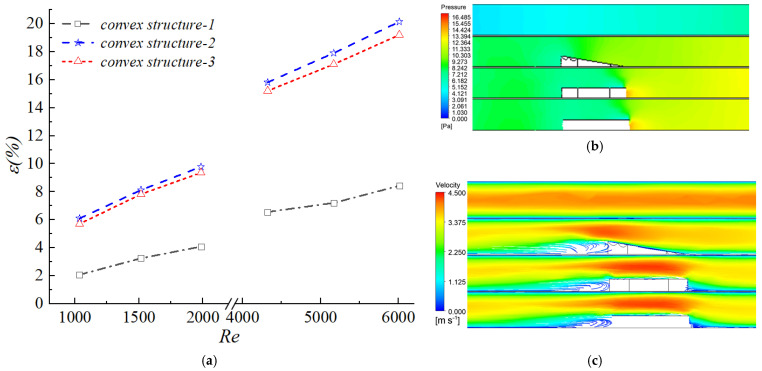
Flow resistance and field characteristics of channels with different convex structures: (**a**) Relationship between pressure drag ratio (*ε*) and Reynolds number (Re); (**b**) Static pressure contours at Re = 1985; (**c**) Velocity streamlines illustrating the influence of different structures.

Figure 8a illustrates the variation of the pressure drag ratio (ε) as a function of the Reynolds number for biomimetic channels with three distinct protuberance structures. In a smooth channel, under both laminar and turbulent regimes, the flow resistance originated entirely from the wall shear stress induced by fluid viscosity, representing pure viscous drag. However, the introduction of protuberances incorporated an additional resistance component: pressure drag.

The underlying mechanism was twofold: first, the protuberances promoted boundary layer transition or enhanced turbulence, thereby increasing the wall shear stress. This led to higher viscous drag coefficients compared to the smooth channel (slightly higher for Re < 2000 and significantly higher for Re > 3500). Second, the obstruction of the incoming flow by the structures generated a high-pressure zone at the leading edge and a low-pressure zone on the leeward side due to flow separation; this pressure differential constituted the pressure drag. The parameter ε represented the proportion of pressure drag within the total friction factor. As Re increased, the inertial effects of the flow intensified, making the contribution of pressure drag increasingly significant. As shown in Figure 8a, the ratio ε gradually increased from 2–6% at Re = 1000 to a maximum of 20% at Re = 6000.

Figure 8b displays the static pressure contours surrounding the three different protuberance structures at Re = 1985. All structures induced significant static pressure variations in their vicinity, which was the direct cause of pressure drag. However, compared to the cylindrical and hexagonal prism structures—which featured vertical sides and sharp edges—the biomimetic protuberance exhibited less pronounced static pressure variations due to its inclined surfaces and biomimetic groove transition design. Quantitatively, the ε value for the biomimetic protuberance was 0.0051, whereas the values for the cylindrical and hexagonal prism structures were 0.0122 and 0.0128, respectively.

Figure 8c further elucidates the underlying causes through streamlines and cross-sectional velocity contours for the different structures at the same Reynolds number, providing a visual representation of the impact on the velocity field. The biomimetic protuberances occupied the flow space, exerting a throttling effect on the airflow. Consequently, the airflow accelerated sharply when passing over the protuberances. Simultaneously, a distinct low-velocity recirculation zone formed near the structures, where the velocity was extremely low, and backflow occurred on the leeward side. A comparison across the three groups revealed that the characteristics of these low-velocity regions differed significantly, which was closely related to the specific geometry of the protuberance structures.

The low-velocity region at the leading edge of the protuberance structures was primarily governed by the flow stagnation effect. When the incident flow encountered a protuberance, fluid kinetic energy was converted into pressure energy, resulting in a sharp decrease in flow velocity near the wall and the formation of a stagnation zone. Due to its streamlined configuration, the biomimetic protuberance effectively guided the incoming flow to bypass the structure smoothly. This reduced the impact of the flow and the leading edge, leading to the smallest stagnation zone in the velocity contours. Consequently, the stagnation effect was mild, the pressure loss was minimized, and the pressure differential across the structure remained the lowest among the three cases. In contrast, the hexagonal prism and cylindrical structures produced significant edge effects due to their vertical frontal geometries. The sharp transitions between the leading edges and the bottom wall caused a violent impact with the incoming flow, leading to rapid stagnation. This resulted in a markedly expanded stagnation zone and a substantially larger high-pressure region at the leading edge. However, the circular cross-section of the cylinder, lacking sharp corners, slightly mitigated the impact compared to the hexagonal prism, rendering its stagnation effect more moderate.

The low-velocity wake region downstream of the structures originated from the coupling of flow separation and turbulent dissipation. As the fluid flowed around the structures, a boundary layer formed on the wall. When the flow passed the trailing edge, the adverse pressure gradient within the channel rose significantly, triggering boundary layer separation and forming a wake structure characterized by recirculating vortices. The intense dissipation of kinetic energy within these vortices caused the local flow velocity to drop far below that of the mainstream, appearing as blue low-velocity zones in the velocity contours. As illustrated in the velocity contours in Figure 5b, the biomimetic structure exhibited the smallest trailing-edge wake region, featuring a simplified vortex structure and relatively higher flow velocities. This indicated weaker kinetic energy dissipation and lower pressure loss.

Consequently, compared to the hexagonal prism and cylindrical structures, the design of the scale-inspired biomimetic structure effectively reduced both the leading-edge stagnation zone and the downstream low-velocity wake region, thereby demonstrating a significantly lower pressure drag.

### 4.3. Comparative Analysis of Heat Transfer Efficiency for Biomimetic Fins with Different Groove Depths

**Figure 9:**
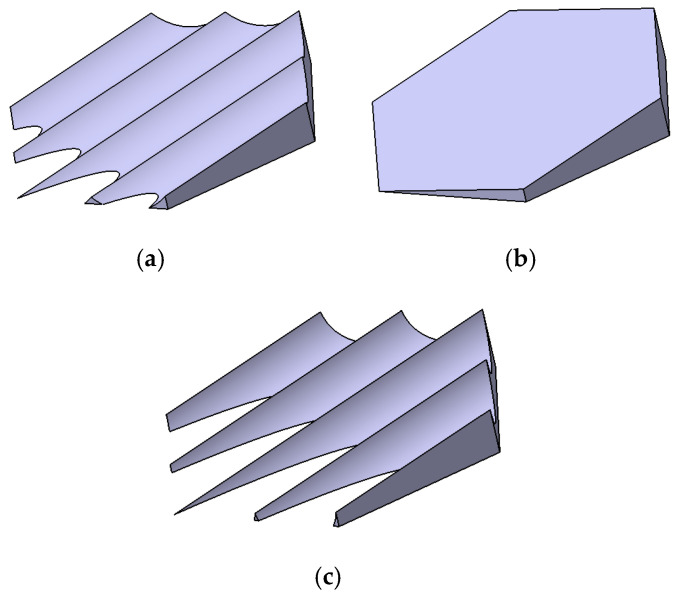
Geometric variants of the biomimetic fin: (**a**) fin with micro-grooves (groove depth = 0.6 mm); (**b**) fin without grooves (smooth sloped surface); (**c**) fin with micro-grooves (groove depth = 1.2 mm).

This section focuses on the biomimetic fins and investigates the influence of the groove depth—a key design parameter inspired by shark skin denticles—on the flow and heat transfer characteristics within the heat exchange unit channels. Through a systematic analysis of the velocity, temperature, and pressure fields, combined with comprehensive indicators such as the Performance Evaluation Criterion (PEC), the heat transfer enhancement mechanisms and flow control effects of these structures under different groove depths were revealed. Furthermore, the study explored the underlying laws governing the impact of structural features on local flow separation, vortex structure evolution, and temperature uniformity. With the groove depth as the primary variable, three variants of the biomimetic fin were designed: a fin with a groove depth of 0.6 mm (Biomimetic Structure 1), a fin without grooves (Biomimetic Structure 2), and a fin with a groove depth of 1.2 mm (Biomimetic Structure 3), as illustrated in Figure 9. Their respective effects on the friction factor (f) and the heat transfer factor (j) were systematically evaluated.

**Figure 10:**
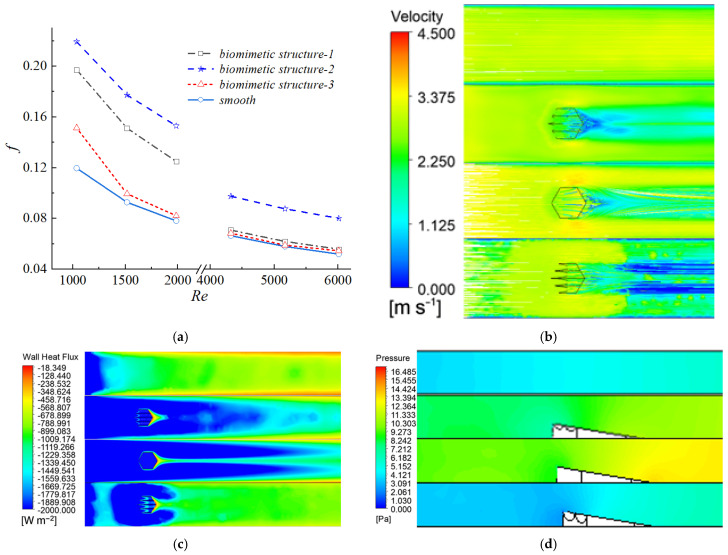
Thermo-hydraulic performance and associated flow field distributions of different biomimetic structures: (**a**) Colburn factor f as a function of Reynolds number for the smooth channel and the three biomimetic fin configurations (Structure 1, 2, 3); (**b**) Velocity contours at the channel cross-section; (**c**) Wall heat flux distributions; (**d**) Pressure field distributions.

Figure 10a illustrates the variation of the friction factor for different biomimetic structures across a range of Reynolds numbers. A comparison of the three structures revealed that Structure 2, which lacked the biomimetic groove design, exhibited the highest flow resistance. At a given Reynolds number, the values of the f factor were found to be positively correlated with the relative cross-sectional area occupied by the structure within the channel; specifically, a smaller cross-sectional occupancy resulted in lower frictional resistance. This trend indicated that the biomimetic grooves effectively reduced flow resistance by optimizing the cross-sectional morphology. Figure 10b displays the cross-sectional velocity contours for the different biomimetic structures at the same Reynolds number, providing a visual representation of their impact on the velocity field. Comparing the three cases, both Biomimetic Structures 1 and 2 exhibited relatively small recirculation zones and rapid velocity recovery. However, due to the absence of grooves in Structure 2, the airflow underwent boundary layer disruption only at the sloped edges, forming a prolonged low-velocity region on the leeward side that triggered significant pressure drag. In contrast, Structure 3 showed the widest recirculation zone and the slowest velocity recovery, suggesting that excessively deep grooves intensified flow separation and hindered effective flow regulation. The wall heat flux contours (Figure 10c) further validated the heat transfer enhancement mechanism. The heat flux downstream of the biomimetic structures was significantly higher than that of the smooth channel. The turbulence perturbations induced by the grooves enhanced fluid mixing, thereby improving both heat transfer uniformity and efficiency. Nevertheless, the excessive depth of the grooves in Structure 3 induced large-scale vortex structures that weakened the near-wall heat transfer capability, indicating the existence of an upper limit for groove depth optimization. Figure 10d presents the pressure field distribution characteristics induced by the different biomimetic structures. Distinct high-pressure stagnation zones were observed at the leading edges of all three structures. Comparatively, Structure 2 exhibited the most pronounced pressure variations, followed by Structure 1, while the pressure fluctuations in Structure 3 were closer to those of a smooth channel. This phenomenon originated from the guiding effect of the grooves on the fluid. The biomimetic protuberances, through groove-induced secondary flows and turbulent perturbations, effectively disrupted the development of the thermal boundary layer and enhanced convective heat transfer between the fluid and the wall. Conversely, the non-grooved structure suffered from more severe boundary layer separation and slow pressure recovery in the wake region due to the lack of flow regulation, leading to a higher proportion of pressure drag. This was consistent with the observed variation in the friction factor f, confirming that the biomimetic grooves achieved effective control over pressure drag by optimizing the pressure distribution.

**Figure 11:**
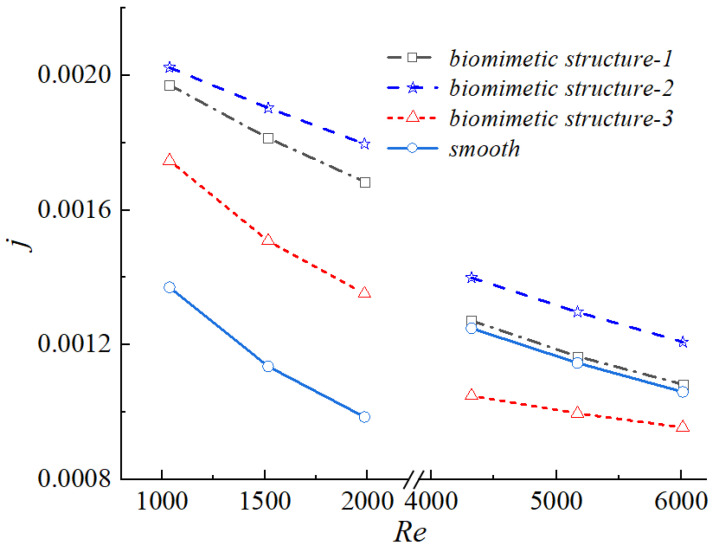
Colburn factor j as a function of Reynolds number for the smooth channel and the three biomimetic fin configurations (Structure 1, 2, 3).

Figure 11 illustrates the variation of the Colburn factor j for different biomimetic structures under identical Reynolds numbers. Among the three configurations, the j factor remained higher than that of the smooth channel over most of the Reynolds number range and exhibited a decreasing trend with increasing Re. However, the rate of decline was slower than that observed in the smooth channel.

Notably, Structure 3 showed a lower j factor than the smooth channel at high Reynolds numbers and also underperformed compared with the other two biomimetic configurations in the low Reynolds number regime. These results indicated that the heat transfer enhancement induced by the biomimetic grooves was effective only within a specific geometric range. Excessively deep grooves disrupted boundary layer stability and weakened near-wall heat transfer, ultimately leading to a deterioration in overall thermal performance.

**Figure 12:**
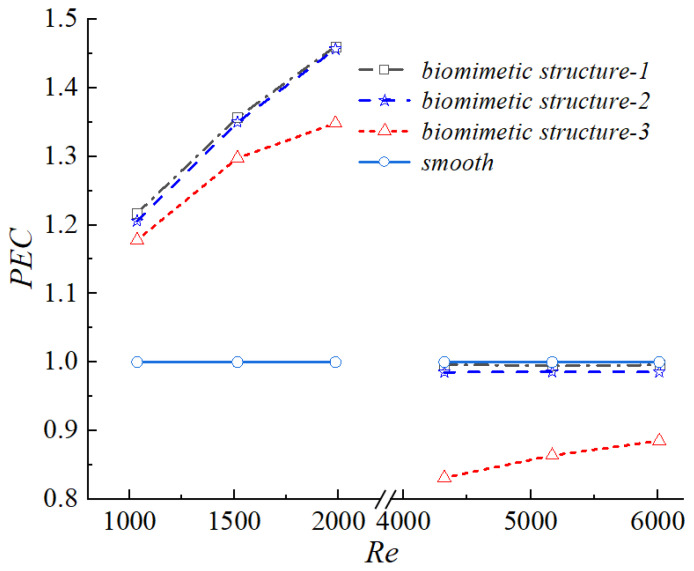
Performance evaluation criterion (PEC) as a function of Reynolds number for the smooth channel and the three biomimetic fin configurations (Structure 1, 2, 3).

Figure 12 presents the variation of the performance evaluation criterion with Reynolds number for the three biomimetic configurations. From a mechanistic perspective, Structure 1 achieved an optimal balance between heat transfer enhancement and flow resistance control by employing an appropriate groove depth. The turbulence perturbations induced by this configuration effectively increased the Colburn factor j, while the groove geometry suppressed excessive boundary layer separation. As a result, the pressure drop and frictional resistance were significantly reduced, and the increase in the friction factor f remained substantially smaller than the enhancement in j. Consequently, Structure 1 maintained the highest PEC value over the entire Reynolds number range.

Although Structure 2 exhibited a relatively high j factor, the absence of an inclined groove surface intensified boundary layer separation. The enlarged separation vortices and recirculation zones markedly increased the pressure drag, leading to a sharp rise in the friction factor f. The increase in flow resistance exceeded the thermal benefit gained from the enhanced heat transfer, resulting in a PEC value lower than that of Structure 1.

For Structure 3, the excessive groove depth caused a loss of control over the near-wall flow: the streamwise vortices generated within the grooves became overly large, inducing premature boundary layer separation and forming a large-scale low-velocity recirculation zone downstream (as shown in Figure 10b). Within this recirculation zone, the fluid temperature tended to become uniform and the thermal boundary layer thickened, resulting in a decrease in local heat flux (as shown in Figure 10c). Although the recirculation zone reduced the pressure drag, it weakened the convective heat transfer between the fluid and the wall, thus failing to effectively enhance heat exchange. Consequently, while the increase in the friction factor f for Structure 3 was smaller than that of the other two configurations, the improvement in the j factor was also significantly lower, leading to the lowest PEC value among the three. This phenomenon indicates that there exists an optimal range for groove depth; excessively deep grooves disrupt the synergy of near-wall flow, making it impossible to simultaneously achieve drag reduction and heat transfer enhancement.

The above results indicated that an optimal range of groove depth existed for the biomimetic configuration. Either excessively shallow or overly deep grooves disrupted the synergistic relationship between heat transfer enhancement and flow resistance control. A groove depth of 0.6 mm achieved the best comprehensive thermo-hydraulic performance by effectively balancing heat transfer improvement and frictional penalty. These findings provided essential theoretical support and parametric guidance for the biomimetic surface design of high-efficiency heat transfer devices.

## 5. Conclusions

This study systematically investigated the thermo-hydraulic performance of biomimetic protrusion structures in a rectangular channel, with particular emphasis on flow mechanisms, resistance characteristics, and the influence of groove depth. The results demonstrated that the introduction of biomimetic protrusions significantly altered the flow structure and thermal boundary layer development, thereby affecting the overall heat transfer and pressure drop characteristics.

It should be noted that the Reynolds number range validated in this study is 1000–7000, covering the laminar to transitional flow regimes. The results indicate that the biomimetic groove structures exhibit optimal comprehensive performance at Reynolds numbers below 2000, with PEC values significantly higher than those of the smooth channel. However, the applicability of these structures at Reynolds numbers above 7000 has not been investigated in the present work. Although preliminary analysis suggests a decreasing trend in PEC with increasing Reynolds number, whether the biomimetic structures can maintain their performance advantages at higher Reynolds numbers, or whether structural optimization could extend their applicable range, remains to be further validated. Future studies combining high-Reynolds-number experiments or large eddy simulations (LES) are recommended to thoroughly explore the performance boundaries of these structures.

Compared with the smooth channel, all protrusion configurations enhanced heat transfer by inducing flow separation, vortex generation, and boundary layer disturbance. In the laminar regime, the heat transfer enhancement was more pronounced due to the promotion of early transition, while the friction penalty was also relatively higher. In the fully turbulent regime, although additional turbulence was generated, the incremental heat transfer benefit gradually approached saturation.

The resistance decomposition analysis revealed that the additional drag introduced by protrusions consisted of both viscous resistance and pressure drag. As the Reynolds number increased, the contribution of pressure drag became increasingly significant. The biomimetic protrusion with streamlined geometry and optimized grooves effectively reduced the size of the upstream stagnation zone and downstream wake region, thereby lowering pressure drag compared with conventional cylindrical and prismatic structures.

Further analysis of different groove depths indicated that groove geometry played a decisive role in balancing heat transfer enhancement and flow resistance. A groove depth of 0.6 mm achieved the optimal synergy between thermal enhancement and resistance control, maintaining the highest performance evaluation criterion (PEC) over the investigated Reynolds number range. In contrast, the configuration without grooves suffered from intensified boundary layer separation and excessive pressure drag, while excessively deep grooves overdisturbed the near-wall flow, leading to premature separation and limited heat transfer improvement.

Overall, the results confirmed that an optimal groove depth range existed for biomimetic surfaces. Proper geometric design could effectively regulate secondary flow structures, suppress excessive separation, and achieve superior comprehensive thermo-hydraulic performance. These findings provide theoretical guidance and structural design parameters for the development of high-efficiency biomimetic heat transfer surfaces.

## Figures and Tables

**Table 1:** Uncertainty Analysis of $T_{\mathrm{out}}$ and Δp.

	Re		1036	1516	1985	4321	5169	6011
Project								
Smooth Channel		Δ*p*(Pa)	6 ± 0.58	9 ± 0.58	13 ± 0.58	38.4 ± 0.89	48.4 ± 1.76	59.2 ± 2.89
		*T_out_*(K)	285.22 ± 0.98	289.36 ± 0.92	293.68 ± 1.01	304.36 ± 1.08	306.38 ± 1.11	308.44 ± 1.15
Biomimetic Channel		Δ*p*(Pa)	6 ± 0.58	10 ± 0.58	14 ± 0.58	41.2 ± 1.72	52.4 ± 2.49	64.2 ± 2.75
		*T_out_*(K)	284.64 ± 1.59	288.48 ± 1.50	291.68 ± 1.74	303.74 ± 1.62	305.64 ± 1.51	307.82 ± 1.60

**Table 2:** Numerical Simulation of $T_{\mathrm{out}}$ and Δp.

	Re		1036	1516	1985	4321	5169	6011
Project								
Smooth Channel		Δ*p*(Pa)	5.28	8.93	12.92	37.66	47.96	59.01
		*T_out_*(K)	287.00	291.25	294.60	305.40	307.85	309.76
Biomimetic Channel		Δ*p*(Pa)	5.45	9.28	13.51	40.16	51.43	63.72
		*T_out_*(K)	286.75	290.89	294.22	305.04	307.51	309.42

## Data Availability

The raw data supporting the conclusions of this article will be made available by the authors on request.
